# Stochastic modeling of phenotypic switching and chemoresistance in cancer cell populations

**DOI:** 10.1038/s41598-019-46926-x

**Published:** 2019-07-26

**Authors:** Niraj Kumar, Gwendolyn M. Cramer, Seyed Alireza Zamani Dahaj, Bala Sundaram, Jonathan P. Celli, Rahul V. Kulkarni

**Affiliations:** 1 Department of Physics, University of Massachusetts Boston, Boston, MA 02125 USA; 2 Present Address: Department of Radiation Oncology, Perelman School of Medicine, University of Pennsylvania, Philadelphia, PA USA; 3 Present Address: School of Physics, Georgia Institute of Technology, Atlanta, GA 30332 USA

**Keywords:** Cancer, Biophysics

## Abstract

Phenotypic heterogeneity in cancer cells is widely observed and is often linked to drug resistance. In several cases, such heterogeneity in drug sensitivity of tumors is driven by stochastic and reversible acquisition of a drug tolerant phenotype by individual cells even in an isogenic population. Accumulating evidence further suggests that cell-fate transitions such as the epithelial to mesenchymal transition (EMT) are associated with drug resistance. In this study, we analyze stochastic models of phenotypic switching to provide a framework for analyzing cell-fate transitions such as EMT as a source of phenotypic variability in drug sensitivity. Motivated by our cell-culture based experimental observations connecting phenotypic switching in EMT and drug resistance, we analyze a coarse-grained model of phenotypic switching between two states in the presence of cytotoxic stress from chemotherapy. We derive analytical results for time-dependent probability distributions that provide insights into the rates of phenotypic switching and characterize initial phenotypic heterogeneity of cancer cells. The results obtained can also shed light on fundamental questions relating to adaptation and selection scenarios in tumor response to cytotoxic therapy.

## Introduction

Acquisition of drug resistance constitutes a major challenge in cancer therapy^1–13^. Therapeutic agents (with widely varying biochemical mechanisms) often exhibit a common pattern of providing an initial reduction in tumor burden followed by recurrence of therapeutically resistant disease with more aggressive progression^6,11,12,14^. Tumor recurrence, which is a major obstacle for cancer cure, is primarily associated with the survival and growth of cell phenotypes that are resistant to chemotherapy^15–17^. Therefore, in order to develop new strategies for the effective treatment of human cancers, a quantitative understanding of the underlying processes leading to drug resistance is essential.

Cellular phenotypic heterogeneity is widely observed in many cancers^3,5,8,18–20^ as a tumor is often composed of multiple subpopulations^21,22^ that show different responses to chemotherapy^9^. In particular, cellular phenotypes that are not sensitive to drugs survive the treatment and can drive drug resistance. As the underlying processes that can lead to the emergence of resistant cells are often stochastic, tumors may locally contain varying numbers of resistant cells. Therefore, quantifying the statistics of drug resistant cells in a tumor is important for effective therapy. Specifically, we are interested in studying population heterogeneity at the start of therapy and aim to address an important issue of therapeutic importance, namely, how to quantify randomness in the numbers of resistant cells prior to drug treatment.

In analyzing population heterogeneity in tumors, a basic question that arises is: How are cell phenotypes that confer survival advantage in the presence of chemotherapeutic drugs generated? A common explanation for the emergence of such phenotypes revolves around Darwinian selection of pre-existing cellular heterogeneity that arises due to random genetic mutations^23–25^. However, the fact that resistant cells switch reversibly to sensitive cells, and that resistant cells often appear on short time intervals (hours to few days), starting from clonal populations, suggests that non-genetic factors play a major role in the generation of phenotypic heterogeneity^5,7,13,26–31^. Such non-genetic phenotypic heterogeneity can arise due to multistability in the underlying gene expression dynamics^32,33^ and noise in gene expression^34–36^. It is interesting to note that non-genetic factors are known to generate phenotypic variation and provide fitness benefits in diverse systems, e.g. in the evolution of microbial colonies under stress^36^. Similar advantages have been reported in the context of *Saccharomyces cerevisiae* wherein phenotypic transitions due to non-genetic factors are known to enhance fitness^37^. Furthermore, theoretical studies have shown how factors such as fluctuations in gene expression can lead to the long-term survival of drug-resistant populations^38^ and how stochastic switching between distinct cellular phenotypes under fluctuating environments can lead to optimal population growth^39,40^.

These observations suggest that there are two distinct, though not mutually exclusive, mechanisms for the onset of drug resistance in cancer cells: (1) cell phenotypes that are resistant to chemotherapy pre-exist in the tumor prior to treatment and are selected for during the treatment, and (2) cells are induced to develop or acquire resistance due to treatment. Thus, it is important to distinguish between selection of pre-existing populations which are inherently less chemosensitive versus adaptive changes in cancer cells that are activated by exposure to chemotherapy. In the cancer biology literature, the latter option is often referred to as adaptation^41^. This adaptation-selection scenario was first explored in the famous Luria-Delbrück experiments^42^ to understand the mechanism of bacterial resistance to bacteriophage infections. The corresponding analysis gave rise to the celebrated fluctuation test which is also used to estimate mutation rates in bacteria. It is important to note that, while in the Luria-Delbrück case phenotypic changes are driven by genetic mutations and thus an irreversible process, in our study, we are considering phenotypic changes that are reversible. Besides reversible phenotypic switching, it is important to consider intrinsic stochasticity in the underlying processes and to characterize cellular heterogeneity as highlighted by previous studies focusing on modeling drug resistance in cancer^43,44^.

In consideration of cellular mechanisms likely to be associated with drug resistance, the epithelial-mesenchymal transition (EMT) emerges as a logical candidate. EMT is a conserved cellular program that enables cells of epithelial lineage to transiently acquire traits of mesenchymal cells, including reversible loss of adherens junctions and gain of proteins associated with enhanced motility, adhesion to extracellular substrates and remodeling of the extracellular matrix^45,46^. In cancer cells, this ability to reversibly adopt a more motile phenotype has been linked to tumor invasion and metastasis^47–49^ but, more importantly for this study, EMT is also directly linked to chemotherapy resistance and cancer stem cell (CSC) properties^50,51^. The mechanisms through which cancer cells having undergone EMT become resistant to cancer therapeutics have been investigated in a number of studies and recently reviewed by Shibue and Weinberg^52^. EMT populations have been shown to be resistant to classical chemotherapy drugs via a combination of decreased apoptotic signal transduction, increased drug efflux (increased ATP binding cassette transporter expression), and decreased cell proliferation. Although other therapeutics are not explored in the present study it is also worth noting that EMT populations also exhibit resistance to molecular targeted and immunotherapy agents through other mechanisms which have also been studied. In the context of this background, experimental studies described herein focus on established markers of epithelial and mesenchymal phenotype in relation to chemotherapy response, which in this report involves pancreatic ductal adenocarcinoma (PDAC) cells. While recognizing that EMT is more likely a spectrum of intermediate states^53–57^, the strong correlation in phenotype and drug response reported here motivates the adoption of two coarse-grained states to be used in the model development. Specifically, a relatively drug-sensitive state with more pronounced epithelial characteristics (E); and a drug-resistant state with increased mesenchymal characteristics (M). In the following sections, we will consider these phenotypes to form the basis of a two-state model of the dynamics of phenotypic switching and associated survival of cancer cells under cytotoxic stress^22,26^.

The paper is organized as follows. We begin with a brief description and results from a set of motivating experiments, in which drug resistance is evaluated as a determinant of phenotype in pancreatic cancer cells *in vitro*, and conversely, phenotype as a determinant of drug response in the same cells. The two phenotype switching model inspired by the experimental data is then described. We then develop an analytic approach to quantify population heterogeneity at the start of therapy followed by a protocol for estimating phenotypic switching parameters. We then construct an approximate approach for characterizing the probability distribution of the fraction of resistant cells in a population. We conclude with remarks on possible extensions and future directions.

## Results

### Evaluation of drug resistance and phenotype in cell culture studies

**Figure 1:**
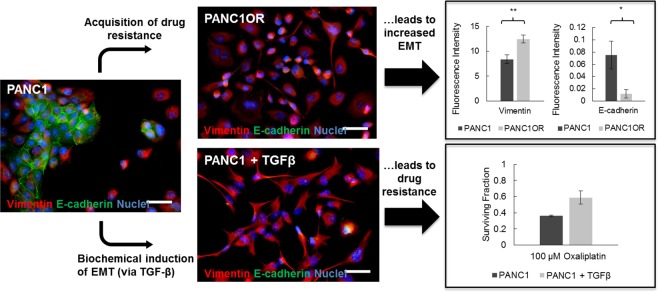
Equivalence in the acquisition of chemotherapy resistance and epithelial-mesenchymal transition in pancreatic cancer cells.

We first sought to compare phenotypic traits in naive and drug-resistant pancreatic ductal adenocarcinoma (PDAC) cells. PANC1 cells (a quasimesnchymal PDAC cell line^58^) were exposed to increasing doses of oxaliplatin chemotherapy over successive passages until resistant cells were stable through multiple passages and cryopreservation. As shown in Fig. 1 (upper panels), acquisition of chemoresistance leads to a marked change in phenotype from naive cells displaying characteristically epithelial adherens junctions, to drug-resistant cells with highly branched morphology, no evident E-cadherin, and marked increase in cytoskeletal vimentin (IF quantification, upper right). This pattern of changes in E-cadherin and vimentin expression are classic and well-established markers of EMT. We further examined the reciprocal scenario, in which the same parental cells were directly induced^45^ to undergo EMT via administration of exogenous TGF-*β* (Fig. 1, lower panels). The resultant phenotype is strikingly similar to that of our drug-resistant cells and importantly, exhibits resistance to chemotherapy similar to when resistance was acquired directly through drug exposure. Collectively these results display a symmetry in that acquisition of drug resistance in epithelial cancer cells leads to increase in mesenchymal characteristics, while direct transition from epithelial to mesenchymal phenotype leads to drug resistance.

### Coarse-grained Model

**Figure 2:**
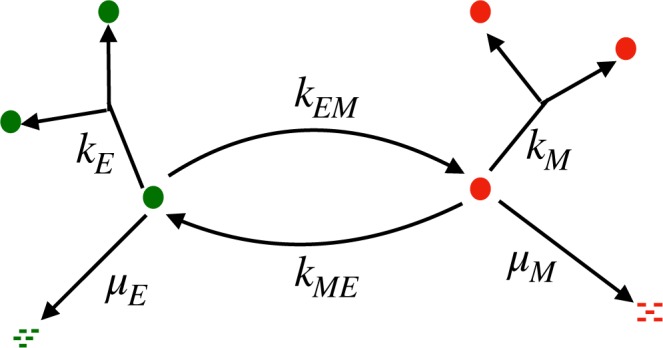
Schematic representation of the two phenotype EMT model of tumor growth: Sensitive and resistant phenotypes are shown as green and red circles respectively. The phenotypic switching rates are represented by *K*_*EM*_ and *K*_*ME*_, birth rates by *k*_*E*_ and *k*_*M*_, and death rates by *μ*_*E*_ and *μ*_*M*_.

Motivated by the preceding observations and by previous work^22,26^, we now consider a simple coarse-grained model (Fig. 2) for phenotypic heterogeneity in tumor cells. We consider that the population of cancer cells consists of two distinct subpopulations; drug-sensitive or drug-tolerant. Based on our experimental results, we denote the drug-sensitive population by *E* (for epithelial phenotype) and the drug-tolerant population by *M* (for mesenchymal phenotype). The processes that control the evolution of tumor heterogeneity are as follows: (1) *birth*: each *E*-type or *M*-type cell gives rise to birth of new cells of the same type with rates *k*_*E*_ and *k*_*M*_, respectively; (2) *death*: each *E*-type (*M*-type) cell degrades with rates *μ*_*E*_ (*μ*_*M*_); (3) *phenotypic switching*: an *E* cell can switch to a *M* cell with rate *k*_*EM*_, and *M* cell can switch back to *E* cell with rate *k*_*ME*_. We assume that intrinsic rates of phenotypic switching and decay of each type of cancer cell are the same throughout the sample.

At any time *t*, the state of the system is defined by the number of *E* and *M* cells. The temporal evolution of the corresponding probability distribution is given by the master equation:

1 $$\begin{array}{rcl}\frac{\partial P(E,M,t)}{\partial t} & = & {k}_{E}(E-1)P(E-1,M,t)+{k}_{M}(M-1)P(E,M-1,t)\\  &  & +\,{\mu }_{E}(E+1)P(E+1,M,t)+{\mu }_{M}(M+1)P(E,M+1,t)\\  &  & +\,{k}_{ME}(M+1)P(E-1,M+1,t)+{k}_{EM}(E+1)P(E+1,M-1,t)\\  &  & -\,[{k}_{E}E+{k}_{M}M+{\mu }_{E}E+{\mu }_{M}M+{k}_{ME}M+{k}_{EM}E]P(E,M,t),\end{array}$$

where *P*(*E*, *M*, *t*) denotes the probability that there are *E* and *M* numbers of epithelial and messenchymal cells present at time *t*.

**Figure 3:**
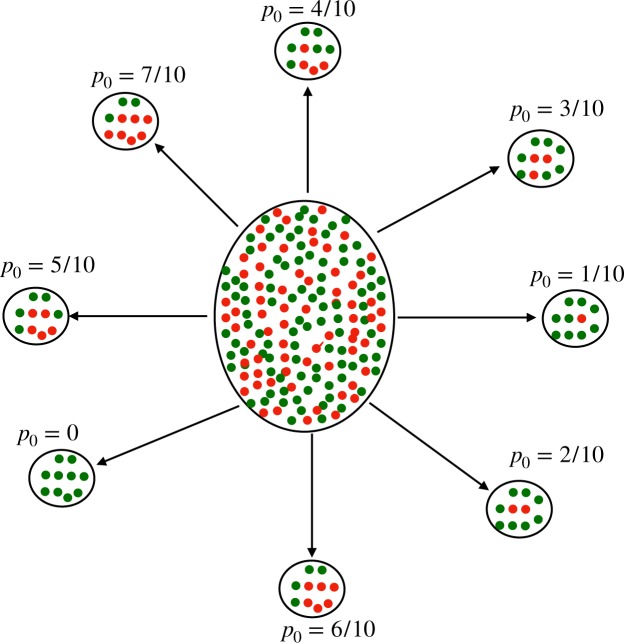
Schematic representation of tumor containing drug-sensitive (green circles) and drug-resistant cells (red circles) is shown at the center. For the sake of conceptual visualization, we have shown different samples taken from the tumor, each characterized by the same number of total cells (here *N*_0_ = 10) but different number of M-cells, and thus different values for *p*_0_ = *M*/*N*_0_.

Within the framework of this model (Fig. 2), we now address a key issue: How to characterize the *initial* heterogeneity (i.e. prior to the start of drug exposure) in tumor cells. Consider a snapshot of the cell population in a tumor composed of distinct spatial regions. Each local neighborhood of the tumor can, in general, have widely different fractions of *M* cells in the local population; this is the heterogeneity we wish to quantify using our model. The proposed protocol for quantifying such heterogeneity involves drawing samples from different spatially distinct regions of the tumor, with each sample corresponding to a fixed number (*N*_0_) of cells, see Fig. 3. Let *p*_0_ = *M*/*N*_0_ denote the local fraction of *M*-cells in the sample population. Thus, the probability (*p*_0_) that a randomly chosen cell in the sample is a *M* cell can itself be considered to be a random variable. We denote the corresponding probability density function by *ρ*(*p*_0_) and characterize it by its mean 〈*p*_0_〉 and variance ${\sigma }_{{p}_{0}}^{2}=\langle {p}_{0}^{2}\rangle -{\langle {p}_{0}\rangle }^{2}$. Thus, initial tumor heterogeneity is characterized not just by the presence of drug-tolerant *M* cells in the sample but also by variations in the number of *M* cells from sample to sample, characterized by the distribution *ρ*(*p*_0_). Note that the sample size *N*_0_ should be large enough such that the sampling noise can be taken to be negligible. Thus the heterogeneity in the local fraction of *M* cells is reflective of the underlying biological heterogeneity rather than sampling noise.

### Analytical results for parameter estimation

We now consider the stochastic process governing evolution of the tumor population upon treatment with drugs. Upon exposure to drugs, it is a reasonable assumption that growth is inhibited, so accordingly we set *k*_*E*_ = *k*_*M*_ = 0. As discussed in the preceding section, we consider the evolution of different sample populations, each of which has a fixed initial size *N*_0_ such that the fraction of *M* cells is drawn from a distribution *ρ*(*p*_0_). In this limit, the key parameters of the model are: ${k}_{EM},{k}_{ME},{\mu }_{E},{\mu }_{M},\langle {p}_{0}\rangle ,{\sigma }_{{p}_{0}}^{2}$. In what follows, we derive analytical results that can be used to estimate model parameters by analyzing the distribution of surviving cells upon drug exposure.

We note that, within our model, the evolution of each cell in the population is independent of the state of the remaining cells. Correspondingly, we first focus on the time evolution of a *single* tumor cell, which is initially either *E*-type or *M*-type with corresponding probabilities as 1 − *p*_0_ and *p*_0_. Let us denote by *P*_*E*_(*P*_*M*_) the probability that the cell is *E*(*M*)-type at time *t*, conditional on the initial probability *p*_0_ for it to be *M*-type. The corresponding probability generating function for the single cell, conditional on the value of *p*_0_ (*g*(*z*_1_, *z*_2_, *t*|*p*_0_) $=\sum _{{\eta }_{E}}\,\sum _{{\eta }_{M}}\,{z}_{1}^{{\eta }_{E}}{z}_{2}^{{\eta }_{M}}P({\eta }_{E},{\eta }_{M},t|{p}_{0})$), can be expressed as

2 $$g({z}_{1},{z}_{2},t|{p}_{0})=1-({P}_{E}+{P}_{M})+{P}_{E}{z}_{1}+{P}_{M}{z}_{2}.$$

It is straightforward to derive analytic expressions for *P*_*E*_(*P*_*M*_) and to thereby obtain an expression for *g*(*z*_1_, *z*_2_, *t*|*p*_0_) (Supplementary Material A). Now, let *G*(*z*_1_, *z*_2_, *t*) denote the probability generating function corresponding to *P*(*E*, *M*, *t*), the probability that we have *E* and *M* number of sensitive (*E*-type) and resistant (*M*-type) cells in the entire population at time *t*. Since each cell in the population (initial size *N*_0_) evolves independently, the probability generating function for the joint distribution at time *t* (averaging over the initial choice of *p*_0_) is given by

3 $$G({z}_{1},{z}_{2},t)={\int }_{{p}_{0}=0}^{{p}_{0}=1}\,d{p}_{0}\rho ({p}_{0})g{({z}_{1},{z}_{2},t|{p}_{0})}^{{N}_{0}},$$

where *ρ*(*p*_0_) is the probability distribution function for the initial fraction of *M*-type cells (*p*_0_).

The expression derived for the generating function, Eq. (3), can be used to derive analytic expressions for all the moments of the marginal distributions corresponding to *E*-type and *M*-type cells at time *t*. For example, expressions for mean number of *E* and *M* cells can be obtained using $\langle E\rangle =dG/d{z}_{1}{|}_{{z}_{1}=1,{z}_{2}=1}\,{\rm{and}}\,\langle M\rangle =dG/d{z}_{2}{|}_{{z}_{1}=1,{z}_{2}=1}$, respectively (see Supplementary Material A). This leads to the following expression for mean number of surviving cells at time *t*, 〈*N*〉 = 〈*E* + *M*〉:

4 $$\begin{array}{rcl}\langle N\rangle /{N}_{0} & = & (\frac{{\gamma }_{0}+{\alpha }_{0}-2({\gamma }_{0}-{\mu }_{E}+{\mu }_{E}{p}_{0}-{\mu }_{M}{p}_{0})}{2{\alpha }_{0}})\exp (-\frac{t}{2}({\gamma }_{0}+{\alpha }_{0}))\\  &  & -\,(\frac{{\gamma }_{0}-{\alpha }_{0}-2({\gamma }_{0}-{\mu }_{E}+{\mu }_{E}{p}_{0}-{\mu }_{M}{p}_{0})}{2{\alpha }_{0}})\exp (-\frac{t}{2}({\gamma }_{0}-{\alpha }_{0})),\end{array}$$

where

5 $${\gamma }_{0}={k}_{EM}+{k}_{ME}+{\mu }_{E}+{\mu }_{M},{\alpha }_{0}=\sqrt{{\gamma }_{0}^{2}-4({k}_{ME}({\mu }_{E}-{\mu }_{M})+({\gamma }_{0}-{\mu }_{M}){\mu }_{M})}.$$

It is clear from the above expression that by fitting the curve corresponding to the mean number of surviving cells as a function of time, the three parameter combinations: *α*_0_, *γ*_0_, and *μ*_*E*_ − *μ*_*E*_*p*_0_ + *μ*_*M*_*p*_0_ can be determined.

To extract the remaining model parameters based on time-course data, we have to turn to analytic results for the higher moments. For example, we can use expressions for the Fano factor (*F*) associated with total number of surviving cells, which is given by $F={\sigma }_{N}^{2}/\langle N\rangle$ with ${\sigma }_{N}^{2}=\langle {N}^{2}\rangle -{\langle N\rangle }^{2}$ denoting the variance in the number of surviving cells. The expression for ${\sigma }_{N}^{2}$ can be obtained using

6 $${\sigma }_{N}^{2}={\sigma }_{E}^{2}+{\sigma }_{M}^{2}+2{C}_{EM},$$

where ${\sigma }_{E}^{2}=\langle {E}^{2}\rangle -{\langle E\rangle }^{2}$ and ${\sigma }_{M}^{2}=\langle {M}^{2}\rangle -{\langle M\rangle }^{2}$ are variances associated with the marginal distributions for the *E* and *M* cells respectively, and *C*_*EM*_ = 〈*EM*〉 − 〈*E*〉〈*M*〉 is the correlation between numbers of *E* and *M* cells. We obtain an explicit expression for the Fano factor given by (see Supplementary Material A):

7 $$F=1-\frac{\langle N\rangle }{{N}_{0}}+\frac{{N}_{0}({N}_{0}-1)}{\langle N\rangle }{[(\frac{({\mu }_{E}-{\mu }_{M})}{{\alpha }_{0}})(\exp (-\frac{t}{2}({\gamma }_{0}-{\alpha }_{0}))-\exp (-\frac{t}{2}({\gamma }_{0}+{\alpha }_{0})))]}^{2}{\sigma }_{{p}_{0}}^{2}.$$

The Fano factor *F* is a measure of deviations from the Poisson distribution, for which *F* = 1. If *F* < 1 or *F* > 1, the distribution is sub-Poissonian or super-Poissonian, respectively. Before turning our attention to approaches for parameter estimation, let us first examine the expression derived for the Fano factor. We note that in the absence of initial variability in the fraction of *M* cells (i.e. ${\sigma }_{{p}_{0}}^{2}=0$), the Fano factor of surviving population is simply given by *F* = 1 − 〈*N*〉/*N*_0_. As the cells are not dividing due to the exposure to drugs, the mean number of surviving cells (〈*N*〉) is less than the initial population of cells (*N*_0_) for *t* > 0. Thus, in this case, the Fano factor is *always* less than one and the distribution of cell population follows a sub-Poissonian distribution. However, given variability in the fraction of *M*-cells in the initial population, the Fano factor can potentially exceed one making the distribution super-Poissonian. This result implies that the observation of a Fano factor in excess of 1 in the distribution of surviving cells is an indicator of variance in the fraction of *M*-cells in the initial population. Thus the measurements of the moments of surviving cell populations can provide evidence for phenotypic heterogeneity in tumor populations prior to drug treatment.

To gain more quantitative insight into the initial heterogeneity, we need to estimate the parameters characterizing the mean and variance of *ρ*(*p*_0_). Let us rewrite Eq. (7) in a more compact form by regrouping terms in the expression to yield the following form

8 $$ {\mathcal F} =(\frac{{\sigma }_{{p}_{0}}({\mu }_{E}-{\mu }_{M})}{{\alpha }_{0}})(\exp (\frac{t}{2}({\gamma }_{0}-{\alpha }_{0}))-\exp (\frac{t}{2}({\gamma }_{0}+{\alpha }_{0}))),$$

where the quantity ${\mathcal F}$ can now be expressed entirely in terms of experimentally measurable quantities such as *F* and 〈*N*〉,

9 $$ {\mathcal F} =\exp [\frac{\mathrm{ln}((F-1+\frac{\langle N\rangle }{{N}_{0}})\frac{\langle N\rangle }{{N}_{0}({N}_{0}-1)})}{2}].$$

Using the expressions for the mean and Fano factor of the surviving population as functions of time, Eqs (4) and (8), we can estimate four of the parameter combinations, namely, *α*_0_, *γ*_0_, *μ*_*E*_ − *μ*_*E*_*p*_0_ + *μ*_*M*_*p*_0_, and $({\sigma }_{{p}_{0}}({\mu }_{E}-{\mu }_{M}))/{\alpha }_{0}$. Correspondingly, we need additional experiments to determine the entire set of 6 model parameters. As we now show, a set of measurements that accomplish this can be obtained by starting from different initial conditions.

The proposed protocol is motivated by that fact that experimental techniques such as fluorescence-activated cell sorting (FACS) can be used to prepare the samples in specified initial states. With this in mind, we begin from an initial condition where all cells are *E*-type i.e. ${p}_{0}=0,{\sigma }_{{p}_{0}}^{2}=0$. Using the derived results, we can determine the parameters *μ*_*E*_, *α*_0_ and *γ*_0_. We next consider the initial condition to be all *M*-type cells i.e. ${p}_{0}=1,{\sigma }_{{p}_{0}}^{2}=0$. Analysis of the corresponding time-course measurements of the number of surviving cells can now be used to estimate the parameter *μ*_*M*_. Having estimated values of *α*_0_, *γ*_0_, *μ*_*E*_, and *μ*_*M*_, we can now find the switching rates, *k*_*EM*_ and *k*_*ME*_, from Eq. (5). Finally, using the expressions for the mean number of surviving cells and corresponding Fano factors for arbitrary *p*_0_, we can get explicit expressions for the probability *p*_0_ and variance ${\sigma }_{{p}_{0}}^{2}$ (Supplementary Material A):

10 $$\begin{array}{rcl}{p}_{0} & = & \frac{\langle N\rangle -{\langle N\rangle }_{0}}{{\langle N\rangle }_{1}-{\langle N\rangle }_{0}},\\ {\sigma }_{{p}_{0}}^{2} & = & \frac{{N}_{0}\langle N\rangle }{{({\langle N\rangle }_{1}-{\langle N\rangle }_{0})}^{2}({N}_{0}-1)}[F-1+\frac{\langle N\rangle }{{N}_{0}}].\end{array}$$

The above results are expressed in terms of experimentally measurable quantities, involving mean values 〈*N*〉_0_ (for *p*_0_ = 0) and 〈*N*〉_1_ (for *p*_0_ = 1) and the Fano-factor of the total surviving population, and thus can be used to estimate the population heterogeneity at the start of drug exposure based on time-course measurements of the surviving population size.

### Modeling generation of tumor heterogeneity

The analysis in the preceding section holds regardless of the source of initial heterogeneity in tumor populations. In this section, we explore how the model introduced for tumor cell dynamics can also be used to analyze a potential mechanism for generation of tumor heterogeneity. We note that the proposed model in Fig. 2 can be seen as a generalized version of the celebrated Luria-Delbrück (LD) model with the important addition that, in the present case, the transition between the two phenotypes is reversible (as opposed to the Luria-Delbrück case). However, while the LD model can be solved exactly^59^, the exact analytical solution of the reversible model in Fig. 2 is not known, to the best of our knowledge. Nevertheless, as we show below, exact expressions for the mean and variance of the number of *E*-type and *M*-type cells can be obtained and used to characterize heterogeneity in tumor cell populations. We can use the master equation, Eq. (1), to derive expressions for the mean number of *E* and *M* cells at any time *t* (Supplementary Material B). Using these expressions, the mean number of surviving cells 〈*N*〉 = 〈*E*〉 + 〈*M*〉 is given by:

11 $$\begin{array}{rcl}\langle N\rangle  & = & (\frac{{E}_{0}(\alpha -\gamma -2{k}_{E}^{f})+{M}_{0}(\alpha -\gamma -2{k}_{M}^{f})}{2\alpha })\exp (-\frac{t}{2}(\gamma +\alpha ))\\  &  & +\,(\frac{{E}_{0}(\alpha +\gamma +2{k}_{E}^{f})+{M}_{0}(\alpha +\gamma +2{k}_{M}^{f})}{2\alpha })\exp (-\frac{t}{2}(\gamma -\alpha )),\end{array}$$

where

12 $$\gamma ={k}_{EM}+{k}_{ME}-{k}_{E}^{f}-{k}_{M}^{f},\alpha =\sqrt{{\gamma }^{2}+4({k}_{ME}({k}_{E}^{f}-{k}_{M}^{f})+(\gamma +{k}_{M}^{f}){k}_{M}^{f})},$$

with ${k}_{E}^{f}={k}_{E}-{\mu }_{E}$ and ${k}_{M}^{f}={k}_{M}-{\mu }_{M}$ representing the effective birth rates for *E*-type and *M*-type cells respectively, while *E*_0_ and *M*_0_ are the initial numbers of *E* and *M* cells at *t* = 0.

The results show that the mean number of surviving cells at any time *t* is characterized by six parameters: initial number of *E* and *M* cells (*E*_0_, *M*_0_), two effective birth rates (${k}_{E}^{f}$, ${k}_{M}^{f}$) and two switching rates (*k*_*EM*_, *k*_*ME*_). Given that the initial population can be chosen in a controlled manner, we can use the results for the mean population size to determine some of the model parameters. Specifically, we can set *M*_0_ = 0 as the initial condition and fitting the data to obtain the coefficient of exponential terms in Eq. (11) will yield ${k}_{E}^{f}$. Next, we can set *E*_0_ = 0 and Eq. (11) will allow us to extract ${k}_{M}^{f}$. Once we estimate ${k}_{E}^{f}$ and ${k}_{M}^{f}$, we can extract the switching rates using the estimated values of *γ* and *α*, using Eq. (12). That is, the proposed procedure allows us to estimate the parameter combinations, ${k}_{E}^{f}$ and ${k}_{M}^{f}$ as well as the parameters *k*_*EM*_ and *k*_*ME*_.

In order to estimate the remaining model parameters, we need to consider the higher moments. While obtaining analytical expressions for the full probability distribution is still an open problem, higher moments can be calculated in a straightforward manner. For example, using Eq. (1) the evolution equation for $\langle {E}^{2}\rangle =\sum {E}^{2}P(E,M,t)$, $\langle {M}^{2}\rangle =\sum {M}^{2}P(E,M,t)$ and $\langle EM\rangle =\sum EMP(E,M,t)$ is given by

13 $$\begin{array}{lll}\frac{{\rm{\partial }}\langle {E}^{2}\rangle }{{\rm{\partial }}t} & = & ({k}_{E}+{\mu }_{E}+{k}_{EM})\langle E\rangle +{k}_{ME}\langle M\rangle +2({k}_{E}-{\mu }_{E}-{k}_{EM})\langle {E}^{2}\rangle +2{k}_{ME}\langle ME\rangle ,\\ \frac{{\rm{\partial }}\langle {M}^{2}\rangle }{{\rm{\partial }}t} & = & ({k}_{M}+{\mu }_{M}+{k}_{ME})\langle M\rangle +{k}_{EM}\langle E\rangle +2({k}_{M}-{\mu }_{M}-{k}_{ME})\langle {M}^{2}\rangle +2{k}_{EM}\langle ME\rangle ,\\ \frac{{\rm{\partial }}\langle ME\rangle }{{\rm{\partial }}t} & = & -{k}_{ME}\langle M\rangle -{k}_{EM}\langle E\rangle +({k}_{E}+{k}_{M}-{\mu }_{E}-{\mu }_{M}-{k}_{ME}-{k}_{EM})\langle ME\rangle +{k}_{ME}\langle {M}^{2}\rangle +{k}_{EM}\langle {E}^{2}\rangle .\end{array}$$

The above set of equations can be solved to get explicit expressions for 〈*E*^2^〉, 〈*M*^2^〉 and 〈*EM*〉 at any time *t* (Supplementary Material B), which can be used to get the variance (${\sigma }_{N}^{2}$) in the total number of surviving cells by using Eq. (6). The analytic expression for the variance, in combination with the expression for mean number of surviving cells, can be used to extract all model parameters. Furthermore, the extracted parameters can then be compared with the parameters derived based on tumor cell dynamics *after* exposure to drugs. The comparisons can provide insight into the relative roles of adaptation and selection in driving tumor heterogeneity. The scenario wherein the switching parameters *k*_*EM*_ and *k*_*ME*_ are effectively unchanged upon exposure to drugs, whereas *μ*_*E*_ and *μ*_*M*_ increase favors selection as the dominant driver of tumor heterogeneity. However, significant changes in the switching rates *k*_*EM*_ and *k*_*ME*_ are indicative of a role for adaptation as well in the generation of tumor heterogeneity.

### Characterizing the distribution of the fraction of resistant cells

The results derived for the moments can also be used to characterize the probability distribution *ρ*(*p*_0_) for the fraction of *M*-type cells. Recall that we must have 0 ≤ *p*_0_ ≤ 1 and furthermore the first two moments of *ρ*(*p*_0_) can be obtained using the procedure outlined in the previous section. Thus a natural choice to characterize the distribution *ρ*(*p*_0_) is to take it to be the Beta distribution with the mean and variance as determined by measurements. The Beta distribution is a natural choice because the domain of a Beta distribution can be viewed as a probability. Another advantage of using a Beta distribution in describing the distribution (or likelihood) of a probability value is that it is a conjugate prior to binomial distribution. This means that, in carrying out Bayesian inference, if the likelihood function is binomial, then a Beta distribution prior will lead to a posterior that is also a Beta distribution (with renormalized parameters). Such a distribution is expressed in terms of two exponents (*α* and *β*) as

14 $$\rho ({p}_{0})=\frac{{\rm{\Gamma }}(\alpha +\beta )}{{\rm{\Gamma }}(\alpha ){\rm{\Gamma }}(\beta )}{p}_{0}^{\alpha -1}{(1-{p}_{0})}^{\beta -1}$$

with mean $\langle {p}_{0}\rangle =\frac{\alpha }{\alpha +\beta }\,{\rm{and}}\,{\rm{variance}}\,{\sigma }_{{p}_{0}}^{2}=\frac{\alpha \beta }{{(\alpha +\beta )}^{2}(\alpha +\beta +1)}.$ The two parameters of the Beta distribution can be estimated using the experimentally determined mean and variance. Explicitly, these are given by

15 $$\alpha =\frac{\langle {p}_{0}\rangle (\langle {p}_{0}\rangle (1-\langle {p}_{0}\rangle )-{\sigma }_{{p}_{0}}^{2})}{{\sigma }_{{p}_{0}}^{2}},\beta =\frac{(\langle {p}_{0}\rangle -1)[{\langle {p}_{0}\rangle }^{2}-\langle {p}_{0}\rangle +{\sigma }_{{p}_{0}}^{2}]}{{\sigma }_{{p}_{0}}^{2}}.$$

**Figure 4:**
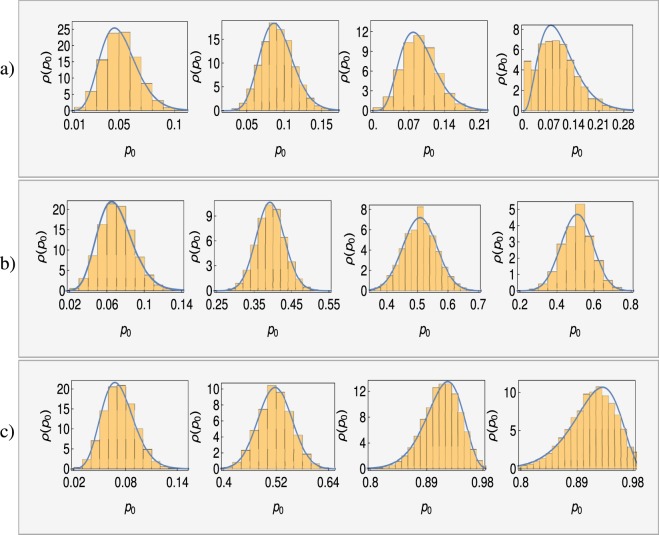
Simulation results for the distributions of fraction of M-cells *ρ*(*p*_0_) are shown as histograms and the continuous solid lines represent fits by Beta distributions. Top (**a**), middle (**b**) and lower (**c**) panels corresponds to *k*_*EM*_/*k*_*ME*_ = 0.1, 1 and 10 respectively. In each panel, distributions are shown at various time points, *t* = 0.1, 1, 10, 20 from left to right. Other parameters are: *k*_*E*_ = 0.2, *k*_*M*_ = 0.1, *μ*_*E*_ = 0.3, *μ*_*M*_ = 0.15.

To test this approach for characterizing the initial heterogeneity, we compare the Beta distribution with the results obtained from stochastic simulations of the model. Specifically, we carried out stochastic simulations using the Gillespie algorithm^60^ for the model in Fig. 2 starting with 200 sensitive E-type cells and no resistant M-type cells. The empirically determined distribution for the fraction of *M*-type cells (*ρ*(*p*_0_)) is then compared to the Beta distribution with the same mean and variance as the empirical distribution. The results obtained are shown in Fig. 4, which indicate that the Beta distribution is an excellent approximation for the range of parameters explored.

## Discussion

To summarize, we have studied a coarse-grained stochastic model to quantify phenotypic heterogeneity in a population of cancer cells. Motivated by the experimental observation that both chemoresistance and TGF-*β* induced EMT lead to similar outcomes, the model assumes that a cell has two phenotypes corresponding to whether it is drug-sensitive or drug-resistant. Importantly, the model is also consistent with epigenetic mechanisms for generating phenotypic heterogeneity in cancer, given that it allows reversible phenotypic switching between sensitive and resistant cells.

For the model considered, we have derived analytic results, both in the presence and absence of chemotherapeutic agents, which provide insights into the role of phenotypic switching in generating population heterogeneity. One of the issues that we address through these results focuses on quantifying initial heterogeneity in the local fraction of resistant cells. This heterogeneity is characterized by mean fraction of resistant cells 〈*p*_0_〉 in a sample and its variance ${\sigma }_{{p}_{0}}^{2}$. We propose a protocol that can be used to estimate the model parameters based on measurements of mean and variance of the surviving population of tumor cells. Furthermore, our analysis also leads to a condition, in terms of experimentally measurable quantities, whose value serves as an indicator for the presence of initial heterogeneity in the fraction of resistant cells.

While the proposed method allows us to estimate the mean and variance of the fraction of resistant cells prior to therapy, obtaining an exact analytical form for the entire distribution appears to be challenging. However, our simulation results suggest that this distribution is well approximated by the Beta distribution, which can be characterized by using the mean and variance of the surviving population. Besides characterizing initial heterogeneity in the cancer cell population, the estimated model parameters can also be useful in analyzing the complex roles of adaptation and selection in the acquisition of chemoresistance. Furthermore, the results obtained provide exact analytical expressions characterizing the distribution of of tumor cell population under treatment by drugs. The simple model considered in this work can serve as a building block for studying models with more explicit spatial dependence. Going forward we envision further model development in dialog with experiments that longitudinally monitor phenotypic changes in time lapse microscopy studies, either by quantitative analysis of morphometric parameters or implementing fluorescent reporters of EMT which have been recently developed^61^. These results can serve as important inputs to future work focusing on evaluation of the hypothesis that model-informed design of treatment schedule and dose parameters may reduce the emergence of chemoresistance.

## Methods

### Cell culture and reagents

PANC1 cells were obtained from the American Type Culture Collection (Manassas, VA), and grown in T-75 cell culture flasks according to ATCC guidelines. DMEM medium (HyClone; Logan, UT) was supplemented with 10% FBS (HyClone; Logan, UT), 100 IU/mL penicillin and 1% streptomycin (HyClone; Logan, UT), and 0.5 ug/mL Amphotericin B (Corning; Corning, NY). The drug-resistant subline, PANC1-OR was generated as described previously^62^. Briefly, increasing concentrations of oxaliplatin were added to each cell type in regular media over successive passages until a stable proliferative phenotype without chemotherapy was observed and maintained following cryopreservation and confirmed by comparative dose response and measurement of a statistically significant increase in IC50.

### Immunofluorescence sample preparation and imaging

Formaldehyde-fixed cells in optical-bottom multiwell plates were incubated overnight at 4 °C with primary antibodies against e-cadherin and vimentin (Cell Signaling EMT Duplex; Danvers, MA). After washing with PBS, cells were incubated for 1 hour with mouse or rabbit Alexa Fluor secondary antibodies (Cell Signaling; Danvers, MA). Cells were mounted with ProLong Gold Antifade reagent containing DAPI (ThermoFisher Scientific Molecular Probes; Waltham, MA) and imaged after 24 hours using a Zeiss LSM 880 confocal microscope with the same detector settings and excitation laser power settings across groups. Images were analyzed using custom Matlab scripts where fluorescent signal for each protein was normalized to the number of cells based on DAPI-stained nuclei.

### Therapeutic response assessment

In sample wells receiving chemotherapy treatment, oxaliplatin (Selleck Chemical; Houston, TX) was added to the media at doses ranging from 0.1 to 500 *μ*M for 48 hours. In experiments where EMT was induced via TGF-beta, 10 ng/mL human recombinant TGF-beta (Gibco, Thermo Fisher Scientific) in 1% FBS DMEM was added to designated wells for 48 hours and respective comparison groups were also grown in 1% FBS for the same duration. In all therapeutic studies treatment conditions were prepared in at least triplicate within each batch including internal controls with sham manipulations. Therapeutic response was assessed via the CellTiter 96® AQueous One Solution Cell Proliferation Assay (Promega; Madison, WI) at 490 nm absorbance in a BioTek® Epoch Microplate Spectrophotometer.

## Supplementary information

Supplementary Material for: Stochastic modeling of phenotypic switching and chemoresistance in cancer cell populations
