# Supplementary material for: Stochastic modeling of phenotypic switching and chemoresistance in cancer cell populations
Source: Sci Rep. 2019 Jul 26;9:10845. doi: 10.1038/s41598-019-46926-x (PMC6659620; doi:10.1038/s41598-019-46926-x)
Supplement: Supplementary file 1 — Supplementary Material for: Stochastic modeling of phenotypic switching and chemoresistance in cancer cell populations [file 41598_2019_46926_MOESM1_ESM.pdf]

Supplementary Material for:
Stochastic modeling of phenotypic switching and chemoresistance in cancer cell populations

Niraj Kumar[1], Gwendolyn M. Cramer[1,†], Seyed Alireza Zamani Dahaj[1,‡], Bala Sundaram[1], Jonathan P. Celli[1] and Rahul V. Kulkarni[1]

[1] *Department of Physics, University of Massachusetts Boston, Boston MA 02125, USA*

[†] *Current address: Department of Radiation Oncology, Perelman School of Medicine, University of Pennsylvania, Philadelphia, PA*

[‡] *Current address: School of Physics, Georgia Institute of Technology, Atlanta GA 30332, USA*

Supplementary Material A: Analytical results for surviving population upon exposure to drugs

Derivation of single cell generating function

Consider a single cell that can exist either as an epithelial(E) or mesenchymal(M) cell. The rates of switching between these two phenotypes (E and M) are given by k_{EM} and k_{ME} and the rates of cell death are given by μ_E and μ_M, respectively. Here we assume no cell divisions (i.e no new production of cells) for either E or M due to high levels of external drugs. For such a system, we consider first the temporal evolution of a single cell, given the initial probability p_0 of the cell being M-type. The corresponding probability generating function is $g(z_1, z_2, t|p_0)$ $= \sum_{\eta_E} \sum_{\eta_M} z_1^{\eta_E} z_2^{\eta_M} P(\eta_E, \eta_M, t|p_0)$, where η_E and η_M can have values 0 or 1, and $P(\eta_E, \eta_M, t|p_0)$ is the probability of having η_E and η_M number of cells at time t, given the initial proability p_0 of the cell being M-type. Clearly $P(1, 1, t|p_0) = 0$ since we are starting with a single cell and no new cells are created. Correspondingly at any time t, we have only three possibilities, either $\eta_E = 1$ and $\eta_M = 0$ or $\eta_E = 0$ and $\eta_M = 1$ or $\eta_E = \eta_M = 0$. Thus we obtain

$$g(z_1, z_2, t|p_0) = P(0, 0, t|p_0) + z_1 P(1, 0, t|p_0) + z_2 P(0, 1, t|p_0). \tag{A1}$$

Denote $P(1, 0, t|p_0) = P_E(t)$, $P(0, 1, t|p_0) = P_M(t)$ and $P(0, 0, t|p_0) = P_0(t)$, and then using the normalization condition, $P_E(t)+P_M(t)+P_0(t) = 1$, we obtain the single particle generating function as derived in the main text, Eq. (2).

To find an explicit expression for the single cell generating function $g(z_1, z_2, t|p_0)$, we need to find expressions for the probabilities $P_E(t)$ and $P_M(t)$. For this, we begin from their evolution equations:

$$\begin{aligned} \frac{dP_E(t)}{dt} &= k_{ME} P_M(t) - (\mu_E + k_{EM}) P_M(t), \\ \frac{dP_M(t)}{dt} &= k_{EM} P_E(t) - (\mu_M + k_{ME}) P_M(t). \end{aligned} \tag{A2}$$

These equations can be solved to give the following expressions for the temporal evolution of the probabilities:

$$\begin{aligned} P_E &= \left[\frac{(1-p_0)(\gamma_0 + \alpha_0 - 2\mu_M) - 2k_{ME}}{2\alpha}\right] \exp\left(-\frac{t}{2}(\gamma_0 + \alpha_0)\right) - \left[\frac{(1-p_0)(\gamma_0 - \alpha_0 - 2\mu_M) - 2k_{ME}}{2\alpha}\right] \exp\left(-\frac{t}{2}(\gamma_0 - \alpha_0)\right), \\ P_M &= \left[\frac{p_0(\gamma_0 + \alpha_0 - 2\mu_E) - 2k_{EM}}{2\alpha}\right] \exp\left(-\frac{t}{2}(\gamma_0 + \alpha_0)\right) - \left[\frac{p_0(\gamma_0 - \alpha_0 - 2\mu_E) - 2k_{EM}}{2\alpha}\right] \exp\left(-\frac{t}{2}(\gamma_0 - \alpha_0)\right), \end{aligned} \tag{A3}$$

where α_0 and γ_0 denote combinations of the model parameters, as given in Eq.(5) in the main text and $p_0 = P_M(t = 0)$ as discussed above. Using the expressions for P_E and P_M from Eq. (A3) in Eq. (2), we obtain the single particle probability generating function.

Derivation of mean and Fano factor for surviving population

Here we provide the details for the derivation of expressions for temporal evolution of moments associated with total surviving population. For this, we start from the expression for the generating function,

$$G(z_1,z_2,t)=\sum_{E=0}^{\infty}\sum_{M=0}^{\infty} z_1^E z_2^M P(E,M,t)=\int_{p_0=0}^{p_0=1} dp_0\rho(p_0)\left[g(z_1,z_2,t)\right]^{N_0}, \tag{A4}$$

and using Eqs.(A4), (2), (A3) and denoting $\langle p_0\rangle = \int p_0\rho(p_0)dp_0$ as the mean value of p_0, arrive at the following expressions for the mean numbers of epithelial and mesenchymal cells,

$$\begin{aligned}\langle E\rangle &= \left.\frac{dG}{dz_1}\right|_{1,1} = N_0(S_0+S_1\langle p_0\rangle),\\ \langle M\rangle &= \left.\frac{dG}{dz_2}\right|_{1,1} = N_0(Q_0+Q_1\langle p_0\rangle),\end{aligned} \tag{A5}$$

where

$$\begin{aligned}Q_0 &= \left(\frac{-2k_{EM}}{2\alpha_0}\right)\left(\exp\left(-\frac{t}{2}(\gamma_0+\alpha_0)\right)-\exp\left(-\frac{t}{2}(\gamma_0-\alpha_0)\right)\right),\\ Q_1 &= \left(\frac{\gamma_0+\alpha_0-2\mu_E}{2\alpha_0}\right)\exp\left(-\frac{t}{2}(\gamma_0+\alpha_0)\right)-\left(\frac{\gamma_0-\alpha_0-2\mu_E}{2\alpha_0}\right)\exp\left(-\frac{t}{2}(\gamma_0-\alpha_0)\right),\\ S_0 &= \left(\frac{\gamma_0+\alpha_0-2(k_{ME}+\mu_M)}{2\alpha_0}\right)\exp\left(-\frac{t}{2}(\gamma_0+\alpha_0)\right)-\left(\frac{\gamma_0-\alpha_0-2(k_{ME}+\mu_M)}{2\alpha_0}\right)\exp\left(-\frac{t}{2}(\gamma_0-\alpha_0)\right),\\ S_1 &= \left(\frac{\gamma_0-\alpha_0-2\mu_M}{2\alpha_0}\right)\exp\left(-\frac{t}{2}(\gamma_0-\alpha_0)\right)-\left(\frac{\gamma_0+\alpha_0-2\mu_M}{2\alpha_0}\right)\exp\left(-\frac{t}{2}(\gamma_0+\alpha_0)\right),\end{aligned} \tag{A6}$$

and α_0 and γ_0 are given by Eq. (5) in the main text. Using Eq.(A5), we see that the mean number of total surviving cells $(E+M)$ is given by

$$\langle N\rangle = N_0\left[Q_0+S_0+(Q_1+S_1)\langle p_0\rangle\right]. \tag{A7}$$

We turn next to the second moment and, using the same generating function, the variances in E and M cells are given by

$$\begin{aligned}\sigma_E^2 &= \left.\frac{d^2G}{dz_1^2}\right|_{1,1}-\left(\left.\frac{dG}{dz_1}\right|_{1,1}\right)^2+\left.\frac{dG}{dz_1}\right|_{1,1},\\ \sigma_M^2 &= \left.\frac{d^2G}{dz_2^2}\right|_{1,1}-\left(\left.\frac{dG}{dz_2}\right|_{1,1}\right)^2+\left.\frac{dG}{dz_2}\right|_{1,1},\end{aligned} \tag{A8}$$

leading to the following expression for the Fano factor:

$$\begin{aligned}F_E &= \frac{\sigma_E^2}{\langle E\rangle} = 1-\frac{\langle E\rangle}{N_0}+\frac{N_0(N_0-1)}{\langle E\rangle}S_1^2\sigma_{p_0}^2,\\ F_M &= \frac{\sigma_M^2}{\langle M\rangle} = 1-\frac{\langle M\rangle}{N_0}+\frac{N_0(N_0-1)}{\langle M\rangle}Q_1^2\sigma_{p_0}^2.\end{aligned} \tag{A9}$$

Substituting the expressions for S_1 and Q_1 and simplifying these Fano factors can be reexpressed as

$$\begin{aligned}F_E &= 1-\frac{\langle E\rangle}{N_0}+\frac{N_0(N_0-1)}{\langle E\rangle}\left[\left(\frac{\gamma_0-\alpha_0-2\mu_M}{2\alpha_0}\right)\exp\left(-\frac{t}{2}(\gamma_0-\alpha_0)\right)-\left(\frac{\gamma_0+\alpha_0-2\mu_M}{2\alpha_0}\right)\exp\left(-\frac{t}{2}(\gamma_0+\alpha_0)\right)\right]^2\sigma_{p_0}^2,\\ F_M &= 1-\frac{\langle M\rangle}{N_0}+\frac{N_0(N_0-1)}{\langle M\rangle}\left[\left(\frac{\gamma_0+\alpha_0-2\mu_M}{2\alpha_0}\right)\exp\left(-\frac{t}{2}(\gamma_0+\alpha_0)\right)-\left(\frac{\gamma_0-\alpha_0-2\mu_M}{2\alpha_0}\right)\exp\left(-\frac{t}{2}(\gamma_0-\alpha_0)\right)\right]^2\sigma_{p_0}^2.\end{aligned} \tag{A10}$$

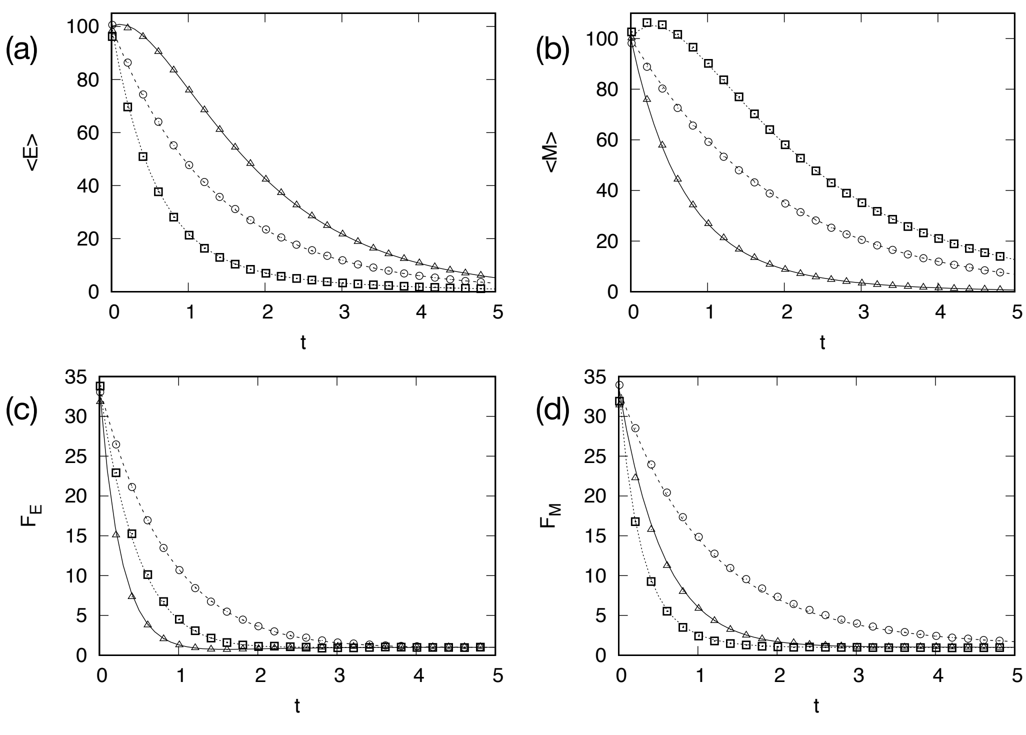

FIG. S1: Temporal variations of the mean and Fano factor for E and M cells: Solid lines are analytic predictions and points correspond to simulation results. The three curves are for different values of k_{EM} and k_{ME}: solid lines ($k_{EM} = 0.1, k_{ME} = 1$), dotted lines ($k_{EM} = 1, k_{ME} = 0.1$), and dashed lines ($k_{EM} = 0.1, k_{ME} = 0.1$), and $\mu_E = 0.75$ and $\mu_M = 0.50$ are same for all the curves. We have taken initial number of cells as 200 and randomness in the population was generated by taking the number of M cells as a uniformly distributed random number between 0 and 200.

Note that both the Fano factors, F_E and F_M, are always less than one if $\sigma^2_{p_0} = 0$. That is, a Fano factor greater than 1 is an indication of the presence of initial variability in the fraction of M cells in the population. The analytical predictions for the mean and Fano factor of the surviving E and M populations are shown in Fig. S1 for a range of parameters, along with results from stochastic simulations using the Gillespie algorithm.

To derive an expression for the variance of the total population $N = E + M$, we use the relation $\sigma^2_N = \sigma^2_E + \sigma^2_M + 2C_{EM}$, where $C_{EM} = \langle EM\rangle - \langle E\rangle\langle M\rangle$ is the correlation between E and M, and $\langle EM\rangle$, in terms of the generating function, is

$$\langle EM\rangle = \frac{d}{dz_1}\left(\frac{dG}{dz_2}\right)\Bigg|_{1,1}. \tag{A11}$$

The expression for the Fano factor for the total population N can be written as

$$F = 1 - \frac{\langle N\rangle}{N_0} + \frac{N_0(N_0-1)}{\langle N\rangle}(S_1+Q_1)^2\sigma^2_{p_0}, \tag{A12}$$

which, on using Eq. (A6), can be rewritten as Eq. (7) in the main text.

Expressions for p_0 and $\sigma^2_{p_0}$

As discussed in the main text, we propose a three-step procedure. First, we set $p_0 = 0$, which, using Eqs. (A7) and (A12), gives

$$\begin{aligned}\langle N\rangle_0 &= N_0(Q_0+S_0),\\ F_0 &= 1-(Q_0+S_0),\end{aligned} \tag{A13}$$

and next, setting $p_0 = 1$,

$$\begin{aligned} \langle N \rangle_1 &= N_0(Q_0 + S_0 + Q_1 + S_1), \\ F_0 &= 1 - (Q_0 + S_0 + Q_1 + S_1). \end{aligned} \tag{A14}$$

Finally, using the expressions for mean number of surviving cells, Eq.(A7), and corresponding expression for the Fano factor, Eq. (A12), for arbitrary p_0, we get explicit expressions for the probability p_0 and variance in initial M-type cells $\sigma^2_{p_0}$. The resulting expressions are in terms of mean values $\langle N \rangle_0$ (for $p_0 = 0$) and $\langle N \rangle_1$ (for $p_0 = 1$) and the mean and Fano factor for a given arbitrary p_0, as shown in the main text.

Supplementary Material B: Analytical results in the growth phase

In this section, we provide details of the derivation of the moments for the surviving cell populations in the growth phase.

First moments

Multiplying Eq. (1) by E or M and summing over all possible values of E and M, we obtain the evolution equations for $\langle E \rangle = \sum EP(E, M, t)$ and $\langle M \rangle = \sum MP(E, M, t)$:

$$\begin{aligned} \frac{\partial \langle E \rangle}{\partial t} &= (k_E - \mu_E - k_{EM}) \langle E \rangle + k_{ME} \langle M \rangle, \\ \frac{\partial \langle M \rangle}{\partial t} &= (k_M - \mu_M - k_{ME}) \langle M \rangle + k_{EM} \langle E \rangle, \end{aligned} \tag{B1}$$

These equations can be solved to get:

$$\begin{aligned} \langle E \rangle &= \frac{1}{2\alpha} \left[(E_0\alpha - \beta_E) \exp\left(-\frac{t}{2}(\gamma + \alpha) \right) + (E_0\alpha + \beta_E) \exp\left(-\frac{t}{2}(\gamma - \alpha) \right) \right], \\ \langle M \rangle &= \frac{1}{2\alpha} \left[(M_0\alpha - \beta_M) \exp\left(-\frac{t}{2}(\gamma + \alpha) \right) + (M_0\alpha + \beta_M) \exp\left(-\frac{t}{2}(\gamma - \alpha) \right) \right], \end{aligned} \tag{B2}$$

where E_0 and M_0 are initial values for the number of E-type and M-type cells respectively, and

$$\begin{aligned} \gamma &= k_{EM} + k_{ME} - k_E^f - k_M^f, \\ \alpha &= \sqrt{\gamma^2 + 4\left(k_{ME}(k_E^f - k_M^f) + (\gamma + k_M^f) k_M^f \right)}, \\ \beta_E &= 2M_0 k_{ME} + E_0 \left(\gamma - 2(k_{EM} - k_E^f) \right), \\ \beta_M &= 2E_0 k_{EM} + M_0 \left(\gamma - 2(k_{ME} - k_M^f) \right) \end{aligned} \tag{B3}$$

with

$$k_E^f = k_E - \mu_E, \quad k_M^f = k_M - \mu_M, \tag{B4}$$

representing the effective birth rates for E-type and M-type cells, respectively. For the total population, $N = E + M$, using Eq. (B2), the mean $\langle N \rangle = \langle E \rangle + \langle M \rangle$ can then be written as:

$$\begin{aligned} \langle N \rangle = & \left(\frac{E_0(\alpha - \gamma - 2k_E^f) + M_0(\alpha - \gamma - 2k_M^f)}{2\alpha} \right) \exp\left(-\frac{t}{2}(\gamma + \alpha) \right) \\ & + \left(\frac{E_0(\alpha + \gamma + 2k_E^f) + M_0(\alpha + \gamma + 2k_M^f)}{2\alpha} \right) \exp\left(-\frac{t}{2}(\gamma - \alpha) \right). \end{aligned} \tag{B5}$$

Second moments

Using Eq. (1), we can write the evolution equation for

$$\langle E^2\rangle = \sum E^2 P(E,M,t), \quad \langle M^2\rangle = \sum M^2 P(E,M,t) \quad \text{and} \quad \langle EM\rangle = \sum EMP(E,M,t)$$

as:

$$\begin{aligned}
\frac{\partial\langle E^2\rangle}{\partial t} &= (k_E+\mu_E+k_{EM})\langle E\rangle + k_{ME}\langle M\rangle + 2(k_E-\mu_E-k_{EM})\langle E^2\rangle + 2k_{ME}\langle ME\rangle, \\
\frac{\partial\langle M^2\rangle}{\partial t} &= (k_M+\mu_M+k_{ME})\langle M\rangle + k_{EM}\langle E\rangle + 2(k_M-\mu_M-k_{ME})\langle M^2\rangle + 2k_{EM}\langle ME\rangle, \\
\frac{\partial\langle ME\rangle}{\partial t} &= -k_{ME}\langle M\rangle - k_{EM}\langle E\rangle + (k_E+k_M-\mu_E-\mu_M-k_{ME}-k_{EM})\langle ME\rangle + k_{ME}\langle M^2\rangle + k_{EM}\langle E^2\rangle. \quad \text{(B6)}
\end{aligned}$$

Eliminating variables in Eq. (B6), we obtain a single ODE for $\langle E^2\rangle$,

$$\frac{d^3\langle E^2\rangle}{dt^3} - c_2\frac{d^2\langle E^2\rangle}{dt^2} + c_1\frac{d\langle E^2\rangle}{dt} + c_0\langle E^2\rangle = \mathcal{K}(t), \quad \text{(B7)}$$

where c_0, c_1 and c_2 are functions of the model parameters

$$\begin{aligned}
c_2 &= 3(k_E+k_M-k_{EM}-k_{ME}-\mu_E-\mu_M), \\
c_1 &= 2\left[k_E^2+k_{EM}^2+k_M^2-2k_Mk_{ME}+k_{ME^2}-4k_M\mu_E+4k_{ME}\mu_e+\mu_E^2-2k_m\mu_M+2k_{ME}\mu_M+4\mu_e\mu_M+\mu_M^2\right. \\
&+ \left. 2k_{EM}(-2k_M+k_{ME}+\mu_E+2\mu_M)-2k_E(k_{EM}-2k_M+2k_{ME}+\mu_E+2\mu_M)\right], \\
c_0 &= 4(k_E+k_M-k_{EM}-k_{ME}-\mu_E-\mu_M)\left[k_{EM}k_M+k_M\mu_E-k_{ME}\mu_E-(k_{EM}+\mu_E)\mu_M+k_E(k_{ME}+\mu_M-k_M)\right], \quad \text{(B8)}
\end{aligned}$$

and $\mathcal{K}(t)$ is a known function in t:

$$\mathcal{K}(t) = \frac{1}{2k_{ME}^2}\left[(k_M+\mu_M+k_{ME}-k_{EM})\langle M\rangle + k_{EM}\left(1-\frac{k_E+\mu_E+k_{EM}}{k_{ME}}\right)\langle E\rangle + 2(k_M-\mu_M-k_ME)g(t) - \frac{dg(t)}{dt}\right], \quad \text{(B9)}$$

with

$$\begin{aligned}
g(t) &= -\frac{1}{2k_{ME}^2}\left[(k_E+\mu_E+k_{EM})\frac{d\langle E\rangle}{dt} + k_{ME}\frac{d\langle M\rangle}{dt}\right] + \langle M\rangle + \frac{k_{EM}}{k_{ME}}\langle E\rangle + \frac{k_E+k_M-\mu_E-\mu_M-k_{ME}-k_{EM}}{2k_{ME}^2} \\
&\quad \left[(k_E+\mu_E+k_{EM})\langle E\rangle + k_{ME}\langle M\rangle\right]. \quad \text{(B10)}
\end{aligned}$$

The solution for $\langle E^2\rangle$ in Eq. (B7) is:

$$\langle E^2\rangle = \sum_{i=1}^{3} s_i\exp[\lambda_i t] + \sum_{i=1}^{3}\frac{\exp[\lambda_i t]}{3\lambda_i^2-2c_2\lambda_i+c_1}\int \mathcal{K}(t)\exp(-\lambda_i t)dt \quad \text{(B11)}$$

where λ_i, $i=1,2,3$ are the roots of

$$\lambda^3 - c_2\lambda^2 + c_1\lambda + c_0 = 0 \quad \text{(B12)}$$

and s_1, s_2, s_3 are constants to be determined from the initial condition. Once we have obtained an expression for $\langle E^2\rangle$, we can readily derive expressions for $\langle M^2\rangle$ and $\langle ME\rangle$:

$$\begin{aligned}
\langle M^2\rangle &= \frac{1}{2k_{ME}^2}\left[\frac{d^2\langle E^2\rangle}{dt^2} + (3k_{EM}+k_{ME}-3k_E-k_M+3\mu_E+\mu_M)\frac{d\langle E^2\rangle}{dt}\right. \\
&+ \left. 2\left((k_E-k_{EM}-\mu_E)(k_E-k_{EM}+k_M-k_{ME}-\mu_E-\mu_M)-k_{EM}k_{ME}\right)\langle E^2\rangle + 2k_{ME}^2 g(t)\right], \\
\langle ME\rangle &= \frac{1}{2k_{ME}}\left[\frac{d\langle E^2\rangle}{dt} - \langle M\rangle k_{ME} - \langle E\rangle(k_E+k_{EM}+\mu_E) - 2(k_E-k_{EM}-\mu_E)\langle E^2\rangle\right]. \quad \text{(B13)}
\end{aligned}$$

Taking suitable initial conditions, we can find the constants s_1, s_2, s_3 that will help specify the temporal evolution of the second moments in terms of the model parameters. The analytic predictions for the first two moments of E and M populations are shown in the Fig.S2 together with corresponding simulation results.

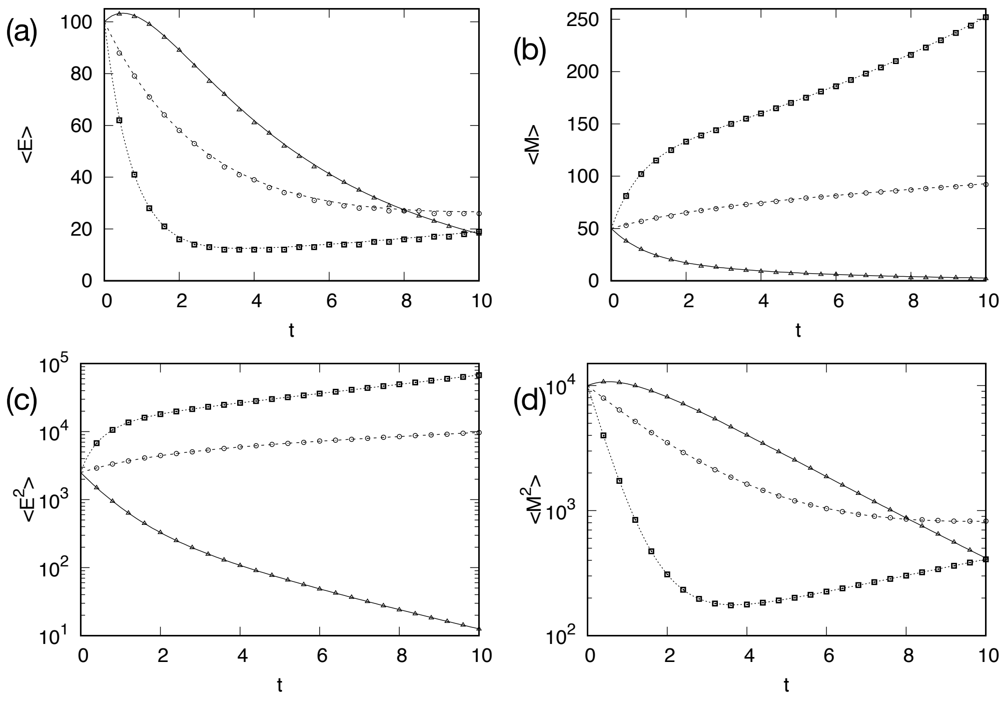

FIG. S2: Temporal variations for the first two moments of E and M populations: Solid lines are analytic predictions and points correspond to simulation results. The three curves are for different values of k_{EM} and k_{ME}: solid lines ($k_{EM} = 0.1, k_{ME} = 1$), dotted lines ($k_{EM} = 1, k_{ME} = 0.1$), and dashed lines ($k_{EM} = 0.1, k_{ME} = 0.1$). Other parameters $k_E = 0.5$, $k_M = 0.6$, $\mu_E = 0.75$ and $\mu_M = 0.50$ are same for all the curves.
